# Revisiting Persistent *Salmonella* Infection and the Carrier State: What Do We Know?

**DOI:** 10.3390/pathogens10101299

**Published:** 2021-10-09

**Authors:** Neil Foster, Ying Tang, Angelo Berchieri, Shizhong Geng, Xinan Jiao, Paul Barrow

**Affiliations:** 1 SRUC Aberdeen Campus, Craibstone Estate, Ferguson Building, Aberdeen AB21 9YA, UK; 2 Institute of Molecular Physiology, Shenzhen Bay Laboratory, Shenzhen 518055, China; tangying@szbl.ac.cn; 3 Departamento de Patologia Veterinária, Faculdade de Ciências Agrárias e Veterinárias, Univ Estadual Paulista, Via de Acesso Paulo Donato Castellane, s/n, 14884-900 Jaboticabal, SP, Brazil; angelo.berchieri@unesp.br; 4 Jiangsu Key Laboratory of Zoonosis, Jiangsu Co-Innovation Center for Prevention and Control of Important Animal Infectious Diseases and Zoonoses, Yangzhou University, Yangzhou 225009, China; gszhong@yzu.edu.cn (S.G.); jiao@yzu.edu.cn (X.J.); 5 School of Veterinary Medicine, University of Surrey, Daphne Jackson Road, Guildford GU2 7AL, UK; paul.barrow@surrey.ac.uk

**Keywords:** *Salmonella*, carrier state, typhoid, immunity, Typhi, Dublin, Pullorum, Gallinarum

## Abstract

One characteristic of the few *Salmonella enterica* serovars that produce typhoid-like infections is that disease-free persistent infection can occur for months or years in a small number of individuals post-convalescence. The bacteria continue to be shed intermittently which is a key component of the epidemiology of these infections. Persistent chronic infection occurs despite high levels of circulating specific IgG. We have reviewed the information on the basis for persistence in *S*. Typhi, *S*. Dublin, *S.* Gallinarum, *S*. Pullorum, *S*. Abortusovis and also *S*. Typhimurium in mice as a model of persistence. Persistence appears to occur in macrophages in the spleen and liver with shedding either from the gall bladder and gut or the reproductive tract. The involvement of host genetic background in defining persistence is clear from studies with the mouse but less so with human and poultry infections. There is increasing evidence that the organisms (i) modulate the host response away from the typical Th1-type response normally associated with immune clearance of an acute infection to Th2-type or an anti-inflammatory response, and that (ii) the bacteria modulate transformation of macrophage from M1 to M2 type. The bacterial factors involved in this are not yet fully understood. There are early indications that it might be possible to remodulate the response back towards a Th1 response by using cytokine therapy.

## 1. Introduction

The majority of *Salmonella enterica* serovars that affect human or animal health generally cause gastrointestinal disease of varying severity in a wide range of hosts but with little systemic disease [1]. In contrast, a small number of serovars are adapted to a narrow range of host species (*S.* Gallinarum, *S*. Pullorum, *S*. Dublin, *S*. Choleraesuis) or specific to one species (*S.* Typhi, *S*. Paratyphi, *S.* Sendai, *S*. Abortusovis and *S*. Abortusequi) and generally produce severe, typhoid-like disease, sometimes with high mortality rates [2]. *S*. Typhimurium and *S*. Enteritidis belong to both groups in that they are the serovars most frequently associated with food poisoning [3], but produce typhoid in susceptible lines of mice [4,5].

**Figure 1:**
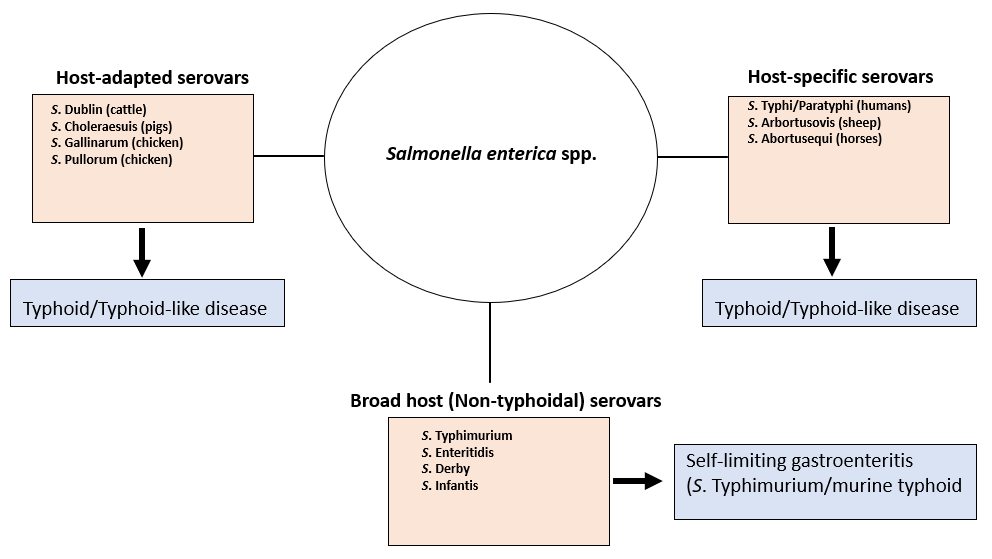
*Salmonella* serovars, host interaction and disease status. *Salmonella enterica* spp. contains circa 1586 serovars which cause disease in warm blooded animals. These can be grouped into host-adapted serovars which although have evolved to adaption within a given host species can infect other hosts (with differential disease outcome), host-specific serovars which only infect a given host species and serovars which can infect a wide range of hosts. Host-adapted and host-specific serovars give rise to typhoid or typhoid-like disease and a percentage of the infected host population will develop asymptomatic persistent infection. Serovars which are neither host-adapted or host-specific can infect a wide range of host species and generally cause gastroenteritis, with the exception of *S*. Typhimurium which causes murine typhoid.

One of the key characteristic features of the typhoid serovars is asymptomatic persistent infections in a proportion of convalescents [6,7,8] (Figure 1). This includes *S*. Typhimurium which also shows chronic, persistent infection in resistant lines of mice.

Given the public health significance of human typhoid and the ready availability of immunological reagents and a detailed knowledge of human and mouse immunology it is not surprising that most progress in understanding persistent infection should be made with both *S*. Typhi and *S*. Typhimurium. Good progress has also been made in understanding persistent *S.* Pullorum infection in domestic fowl. The problems of experimental work with large animals limit the extent to which we understand fully the pathological events that lead to long term persistent carriage in *S*. Dublin and *S*. Abortusovis. This review attempts to bring together currently available information on our current understanding of the basis of persistent infection in five major *Salmonella* serovars, namely *S.* Typhi, *S.* Dublin, *S.* Abortusovis, *S.* Gallinarum and *S.* Pullorum. To avoid confusion, here we use the term persistent infection rather than carrier state which is frequently also used for intestinal carriage.

## 2. What Is the Public Health and Economic Significance of These Diseases?

*S.* Typhi, as the major cause of human typhoid, is responsible for 20 million cases [9,10] and 220,000 deaths worldwide, with a mortality rate of 12–30% [11]. It is now mainly restricted to South East Asia, Africa and South America [12,13,14]. Antimicrobial resistance (AMR) is an increasingly serious problem with >60% strains resistant to the 4 most frequently used antibiotics [15,16,17]. 

*S.* Dublin is a major cause of morbidity, mortality and production losses in dairy cattle. It is the serovar most frequently isolated from cattle with prevalence values between 16–73% of US dairy farms [18] which may vary depending on co-infection with *Fasciola hepatica*. Losses reflect the level of management, herd size, diet and the extent to which herds mix [19,20]. *S*. Dublin is also zoonotic, producing a rare but severe disease with a high mortality rate in humans [6]. 

*S.* Gallinarum and a closely related serovar, *S*. Pullorum, are major problems in countries where high ambient temperatures and limited resources restrict the potential for control by biosecurity. Quantitative economic data are difficult to obtain [21] but mortality rates can reach 90% depending on strain and the genetic background of poultry [22].

*S*. Abortusovis is the serotype most frequently associated with ovine salmonellosis and remains an important cause of economic loss in sheep-rearing countries and especially in Europe and the Middle East [23,24]. The main manifestation is abortion resulting in important economic losses in regions that depend on sheepherding [25,26]. In areas in which the microorganism is endemic, abortion may occur in up to 50% of the ewes in a flock, usually during the first pregnancy [23]. 

## 3. Are There Similarities and Trends in the Clinical Pictures in Acute Infections Produced by These Serovars?

### 3.1. S. Typhi, S. Gallinarum and S. Pullorum

*S*. Typhi and *S.* Gallinarum show very similar courses of acute infection [6,27,28]. Both involve oral infection, invasion from the alimentary tract, probably via M cells [29] and lymphoid tissue including the Peyer’s Patch and, in poultry, the caecal tonsil [30]. Translocation then occurs from the mesenteric lymph nodes (in man) or directly by haematogenous spread (in poultry) to the liver, spleen, bone marrow and gall bladder [6,31,32]. Multiplication occurs in the monocyte-macrophage cell types followed, during clinical disease, by dispersal to lymphoid tissue in the small intestine from where they are excreted in the faeces. This leads to occasional intestinal perforation near the ileo-caecal junction in humans [33,34,35,36], while urinary tract infection can also occur but is less frequent. In human typhoid the gall bladder is frequently infected and is associated with faecal shedding.

*S*. Pullorum is closely related phylogenetically to *S*. Gallinarum but, unlike *S.* Gallinarum, which can infect birds of all ages, *S*. Pullorum generally produces systemic disease only in birds which are no more than a few days old [22]. 

### 3.2. S. Typhimurium 

*S.* Typhimurium also produces a typical clinical picture of typhoid which largely mirrors the typhoid infections of man and poultry above. Unlike *S*. Typhi and *S*. Gallinarum, *S*. Typhimurium colonises the upper alimentary tract of mice well (Barrow, unpublished). Invasion of epithelial cells occurs via a *Salmonella* pathogenicity island (SPI) 1-(SPI-1) mediated process [37] with an inflammatory response. The bacteria are taken up by M cells in the Peyer’s patch in the ileum and multiply within and are disseminated by CD-18-expressing macrophages [38] to the lymph nodes and also eventually the spleen and liver [39]. Macrophage invasion involves phagocytosis, although SPI-1 proteins may also contribute [40]. Macrophages are central to control of *Salmonella* through granuloma formation [41]. The bacteria may proliferate within the *Salmonella*-containing vacuole (SCV) through the activity of proteins encoded by genes found in *Salmonella* pathogenicity island-2 [42,43] and which may lead to cell death [44] or may induce pro-inflammatory mediators [45] which can lead to bacterial killing [44,46]. Control of *Salmonella* in macrophages is dynamic with the involvement of reactive oxidative species in acute infections and reactive nitrosative species in more chronic infections [46].

Immunological control of infection requires cellular and antibody responses [47,48]. The production of IFNγ by NK cells or by CD8 T cells is important in early host-protection. Clearance of the infection requires CD4 T cells and appears to be independent of IFNγ. [49,50].

### 3.3. S. Dublin and S. Abortusovis

The course of acute infection produced by *S.* Dublin and *S.* Abortusovis involves severe enteritis and dysentery in young cattle (calves) and sheep (lambs), respectively, with pneumonia and localisation in the joints in young animals and septicaemia in adult animals [51,52]. 

Following oral infection, *S.* Dublin bacteria are transmitted, mainly extracellularly, to the mesenteric lymph nodes [53] where they may remain but with frequent further dissemination to the spleen and liver, which may result in septicaemia and death depending on host genetic susceptibility. In adult cattle abortion may also be the only sign of infection [54], with still birth or the birth of infected calves. Diarrhoea at the time of parturition causes extensive environmental contamination of the environment resulting in infection of neonatal calves [19,55]. Calf mortality may be as high as 30% [56,57] and colostrum does protect against disease [58]. 

Infection with *S*. Abortusovis is less well studied but from clinical field observations and from experimental work [59] the course of infection and transmission strategy appears to be similar to that of *S*. Dublin with systemic infection in ewes leading to abortion and environmental dissemination with a proportion of lambs surviving and infecting other lambs and ewes. Sanchlis et al. [60] reported that following experimental infection of 18 pregnant ewes; 8 aborted, 4 produced still born lambs and 6 lambs were clinically normal and survived. Heavy discharge from the vagina of infected ewes, due to infected foetuses and placentae, are major causes of infection for a flock. The epidemiology, involving persistence in the adult with cross infection with enteritis in new-born animals, may be important in countries where sheep farming is periodically nomadic [61].

## 4. How Is Short- and Long-Term Carriage/Persistence Manifested?

Long-term persistent infection may be a key evolutionary adaptation of these pathogens to accommodate small host population numbers, for example, in man and Jungle Fowl, the ancestor all chickens and sheep in nomadic societies. Persistent infection with regular/intermittent shedding is a common feature for several other bacterial pathogens including *Mycobacterium tuberculosis*, *Borrelia recurrentis* and several helminth and protozoan parasites [62]. 

### 4.1. S. Typhi

Most human typhoid convalescents continue to shed *S*. Typhi in faeces for <4 weeks with 2–3% becoming long-term persistent carriers which may last for decades [6,63,64,65]. However, in 25% of carriers there is no evidence of acute infection [5,66,67] and a serological study on the presence of Vi antibodies in the Indian population suggests that 10% of the apparently healthy population may be carriers [68]. There is a higher rate of carriage in women [69,70], reflected in transcriptional analysis of blood samples from carriers indicating X and Y chromosome associations [71], and an increased rate of carriage with age [6].

### 4.2. S. Gallinarum and S. Pullorum

In poultry, persistent infection of these two serovars occurs, in the presence of high titre circulating specific IgY, until sexual maturity leads to vertical transmission, via the egg, with horizontal dissemination amongst progeny. Following convalescence from acute infection, *S*. Gallinarum may be shed in the faeces for a period of weeks [27] with long term persistence demonstrable in more resistant lines of birds beyond 14 weeks post-infection [72]. Long term persistence in *S*. Pullorum has been demonstrated experimentally in a susceptible line of chickens, with 20% of birds showing gradually decreasing bacterial numbers in the spleen and liver with clearance between 20–25 weeks of age. In females the decline is interrupted by the onset of sexual maturity which leads to reduced T cell responsiveness [73], resumption of bacterial multiplication in liver and spleen and dissemination to the ovary resulting in 10% of eggs being infected [74]. 

### 4.3. S. Dublin

Adult cows and calves may continue to excrete *S*. Dublin for 4-12 weeks after clinical disease [18] with a smaller number excreting for several years [57,75,76,77,78,79]. It has been estimated that about 5% of convalescent cows become carriers. Long-term shedding may occur from the gut or also in the milk [18,57] but cystitis may also occur with shedding in the urine [56]. Neither age nor breed appear to determine the likelihood of persistence [54] although it occurs most frequently in heifers between the first year of life and 1st calving and abortion tends to occur most frequently in the second half of pregnancy and on farms where infection rates are highest [19]. *S*. Dublin typically shows persistence in Friesian/Holstein cattle breeds which are generally regarded as more susceptible to *Salmonella* infections (C. Wray pers. comm). Epidemiological data from persistently infected farms suggests that persistence occurs in convalescent calves and adult disease may occur either as a result of recrudescence of an asymptomatic persistent infection or from a fulminant primary infection at parturition [75,76,77,80,81]. 

### 4.4. S. Abortusovis

In sheep infected with *S*. Arbortusovis persistent faecal shedding may occur for between 3 and 12 months following abortion [7,82]. How infected lambs show long-term persistence is unclear since most die within a few weeks of birth.

### 4.5. S. Typhimurium

Persistent *Salmonella* infection can occur in mice under different conditions. Inoculation of susceptible BALB/c (*Slc11a1*, also known as *Nramp1*) mice with certain attenuated mutants of *S*. Typhimurium [83,84] or resistant *Slc11a1*^+/+^ mice with fully virulent strains [4,85] have been shown to result in persistence in the tissues for in excess of 70 days [83] and >365 days with faecal shedding for in excess of 180 days [4]. Non-typhoid *Salmonella* serovars also show persistent intermittent faecal excretion [86] but is poorly understood in this context.

**Conclusion:** Short- and Long-term carriage/persistence and dissemination are probably different phenomena with the former occurring during convalescence from acute infections whereas long-term persistence represents a truly chronic infection. Long-term chronic infection seems to occur in two situations (i) with highly virulent bacterial strains and more resistant host phenotypes, and (ii) less virulent bacteria and more susceptible host phenotypes.

## 5. What Is/Are the Main Site(s) of Carriage and Dissemination?

The easy identification of small intestinal lesions in human and fowl typhoid suggests that short term persistence and dissemination undoubtedly involves shedding from these lesions [27,87].

From early work on human typhoid, the spleen, liver and gall bladder have been identified as the main sites for long-term persistence with the latter directly involved in faecal shedding. Ninety percent of carriers have gallstones compared with 25% in the non-infected population [16,88] and there is a further association between gall stones, typhoid and cancer of the gall bladder [89,90,91,92,93,94]. These hepatobiliary cancers have been suggested to result in part from Matrix Metallo-proteinase activity, which is associated with various types of cancer [95,96,97]. There is also a tendency for *S.* Paratyphi to localise in the gall bladder [98].

Typhoid bacilli localised in the gall bladder are known to be more refractory to chemotherapy which is also thought to be related to biofilm production around the gall stone [97,98,99,100,101,102,103]. It may be significant that 80–90% of gall stones show evidence of a biofilm [101]. The gall bladder may also be associated with persistent infection in unusual situations. For example, persistent faecal shedding of a rough mutant of a smooth, virulent *S*. Choleraesuis has been reported from an infected gall bladder in chickens despite this serovar neither colonising nor being virulent for chickens [104]. 

The role of the gall bladder in human typhoid is complicated by the fact that surgical removal (cholecystectomy) does not eliminate shedding completely, indicating the involvement of other organs [105,106,107,108]. *Salmonella* organisms are thought to translocate to the gall bladder either via blood or via the hepatic ducts [2,66]. A study in India of people, who died from causes other than typhoid, found evidence of *S*. Typhi DNA in 8.2% of corpses, 85.7% of which were liver positive as opposed to 42.9% which were positive for *S*. Typhi in the gallbladder and bile [70]. The main sites of persistence of *S*. Gallinarum and *S*. Pullorum are also the liver and spleen but with additional isolation also from the immature ovary and oviduct in birds a few weeks of age [74].

Less information is available on tissue localisation during carriage of *S*. Dublin or *S.* Abortusovis. Sojka et al. [78] reported moderately heavy excretion (Log 4–5 /g faeces) for 2.5 years with isolation at postmortem from the liver, spleen, hepatic lymph node and gall bladder (Log 4–7/gm). In cattle, Lawson et al., [109] reported one persistently shedding calf with an infected gall bladder until adulthood at one year of age.

During persistent infection of mice with attenuated or virulent strains of *S*. Typhimurium, the liver, spleen and mesenteric lymph nodes are colonised with splenomegaly [4,83,84,85], with occasional isolation from the gall bladder [4,84]. Invasion of gall bladder epithelial cells involving SPI-1 [110] is also thought to take place with an inflammatory response and neutrophil infiltration [111].

**Conclusion:** Long-term persistence involves the gallbladder in addition to the liver and spleen.

## 6. Is There Anything Unique to These Serovars That Predisposes Them to Persistence?

All of the 5 serovars covered by this review have become adapted to their hosts and niche with genome shrinkage and accumulation of pseudogenes. In comparison with *S*. Typhimurium, *S.* Typhi has more than 210 pseudogenes, in addition to deletions [112] and, in comparison with *S.* Enteritidis, *S*. Dublin, *S*. Gallinarum and *S*. Pullorum have 82–87, 212–240 and 231–263 pseudogenes, respectively, depending on strain [113,114,115]. It is interesting that of the two avian serovars, *S.* Pullorum, which is less virulent and, could be argued, more closely adapted to the host, has more pseudogenes than *S*. Gallinarum. This serovar also has pseudogenes in the mismatch repair genes *mutH* and *mutL* indicating a greater inherent rate of evolution [116]. The full analysis of the *S.* Abortusovis genome sequence [117] has not yet been published.

Persistence of *S*. Typhi in the gall bladder is associated with neutral mutations and a process of pseudogenisation [118]. These occur randomly and more than one haplotype may occur simultaneously in an individual [119]. Duy et al. [120] similarly found that *S.* Typhi isolated from chronically infected gall bladders showed higher genetic variability compared with acute isolates. This included nonsense mutations affecting a number of physiological characteristics including LPS and the Vi antigen. Neutral evolution reflects the carrier state with rapid transmission of phenotypic changes occurring during acute infections [121]. As with *S.* Typhi it is thought that the high mutation rate in *S.* Gallinarum occurs during persistent infection [116,122] and that this is still occurring with the appearance of new types since 2001. Genetic drift is thought to occur in small populations [112] with no evidence of immune selection. 

*S.* Typhi, *S.* Gallinarum, *S.* Pullorum and *S.* Dublin all have different pseudogenes in the degradation pathway of D-glucarate [114]. Additional pseudogenes occur in the *S.* Typhi, *S.* Gallinarum and *S*. Pullorum operons associated with utilisation of carbon sources ethanolamine, 1,2 propanediol together with anaerobic electron acceptors such as tetrathionate and thiosulphate and cobalamin synthesis [116,123,124] but which are thought to be involved in intestinal metabolism and therefore reflect the poor ability of these three serovars to colonise the intestine in comparison with serovars such as Typhimurium and Enteritidis. A number of fimbrial operons are also not functional in *S*. Typhi, *S*. Dublin and *S*. Gallinarum [114,116,122]. This is complemented by the study of Lawley et al., [125] who found that *fim, csg, pag* and *bpf* fimbria were required in *S*. Typhimurium to persist in mice for up to 28 days. A further time point 49 days was studied but with too few bacteria recoverable for such an analysis to be meaningful.

It is hardly surprising that genes required for intra-cellular survival are also required for persistence. These include the *spv* operon, the SPI-2 pathogenicity Island [125,126] and SPI-1 [125] although Jones et al., [126] found this to be less important for *S.* Gallinarum. Certain auxotrophic attenuations of *S*. Typhimurium, *purA* and *purE,* lead to persistence in the spleen for 10–12 weeks [83]. A mildly attenuated mutant not producing AgfA fibres was also shown to persist for >60 d [84] but the significance of these studies is unclear as attenuation did lead to slower microbial growth and it is unclear whether such a simplistic explanation would be sufficient to explain persistence per se. Resistance to intracellular defensins involving Mig-14 also contributes to long term survival in *S.* Typhimurium [127] and ClpXP protease also contributes to virulence and persistence [128,129].

There does not appear to be any genomic evidence indicating that strains of *S*. Typhi associated with carriers are different to those in the wider population with both showing considerable variation in the core genome [112,130]. There also seems to be no association between phylogeny and persistence in *S*. Dublin [130,131]. Historically, *S.* Pullorum strains have existed in four major lineages [132] with two currently predominating [133] and with new CRISPR types appearing since 2001, this suggests continued evolution. However, there appears to be no clear change in virulence in strains between the 1960s and 2010 [134] and no association between any one clade and persistence.

**Conclusion**: It is difficult to differentiate between the contribution of genomic structure and occurrence of pseudogenes to intracellular infection per se and long-term persistence in the 5 serovars. Neither is there any strong evidence that strains associated with persistence are any different to the wider serovar population.

## 7. Is There a Clear Host Genetic Element That Contributes to the Development of the Carrier State?

To date, very little is known about how the host genetic background influences resistance or susceptibility to persistent as opposed to acute infection. 

Several studies have initiated identification of host chromosomal loci responsible for resistance to acute *S*. Typhi in man [135,136,137,138], *S.* Typhimurium in mice [139] and *Salmonella* in chickens [140,141].

Humans are genetically very outbred such that considerable variation would be expected in the factors which drive establishment of the carrier state in typhoid. Genetic and immunological parameters may, therefore, exist that differentiate the 3% of the population which become carriers from the remainder of the population. In a susceptible commercial chicken line only 20% of birds show persistence of *S.* Pullorum [74] suggesting a bottleneck/stochastic selection or a genetic element to persistence although this has not been explored. 

Slc11a1 is not associated with typhoid susceptibility in man [138] although it, aided by other host genes, is a prerequisite for establishing the carrier state in mice with virulent strains of *S*. Typhimurium [4,85,142]. Similarly, persistence of *S*. Gallinarum only occurs in SAL1-resistant in-bred chickens while in SAL1-sensitive birds the outcome is either fulminant disease or no infection, depending on dose, and with no persistence at all [72]. 

**Conclusion:** The genetic basis of host susceptibility to acute *Salmonella* typhoid-like infections does not appear to reflect the genetic basis of chronic persistent infection and little is known of the basis to the latter.

## 8. Is There Anything Characteristic to the Immune Response during Persistent Infections? 

Much of our understanding of the host response to acute typhoid is derived from murine studies with *S.* Typhimurium. These have indicated the critical role of CD4^+^ Th1 lymphocytes and IL-12 in controlling acute infections in the liver and spleen [143]. IL-12 alone can drive Th1 cell differentiation [144], and this effect is synergized when both IL-12 and IL-18 are activated, to induce Th1 production of IFNγ [144,145]. Similar but less detailed studies have been carried out in chickens infected with *S.* Typhimurium and *S.* Enteritidis with similar responses described [73,146,147,148,149]. 

In the mouse, Th1-directed IFNγ is important in stimulating antibacterial activity in macrophages with reactive oxygen species (ROS) more important in the earlier stages and reactive nitrogen species (RNS) pathways activated in the later (chronic) phase of infection [46]. A study by Hulme et al. [150] also reported that in J774 murine macrophage cells, only typhoid *Salmonella* serovars inhibited RNS pathways; this was associated with wild type *phoP* regulon genes but was prevented in the presence of IFNγ. This indicates that an early survival advantage (prior to the development of a robust IFNγ response following CD4 T lymphocyte infiltration into the intestine) may facilitate dissemination of typhoidal serovars to the deeper tissues.

Much less is known of the characteristics of the immune response to the typhoid *Salmonella* serovars during persistent infection and most of this again comes from work with *S*. Typhimurium in the mouse and more recently with *S*. Pullorum and *S*. Gallinarum in the chicken. From these studies, evidence has accumulated to indicate that the pathogens are able to modulate the host response seen during clearance of acute infection to facilitate persistence with minimal host damage which might arise from a continued inflammatory response [151].

Studies using avian or murine typhoid serovars can be performed relatively easily and such studies may lead to an improved understanding of persistence of other serovars such as *S*. Typhi, *S.* Dublin and *S*. Abortusovis.

### 8.1. S. Typhimurium

**Figure 2:**
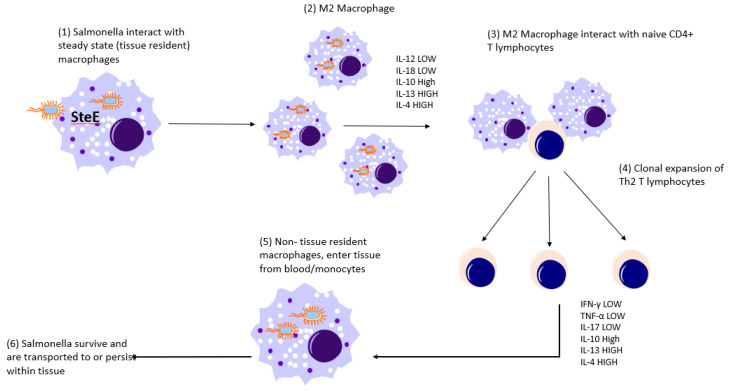
A putative role for the induction of M2 macrophages by *Salmonella* in the induction of persistent infection and carrier status. (**1**) Typhoid *Salmonellae* invade and/or are phagocytosed by resident ‘steady state’ macrophages and secrete effector proteins such as SteE. (**2**) These cause differentiation of anti-inflammatory (M2) macrophages. Which produce low levels of cytokines required for differentiation of Th1 lymphocytes (IL-12 and IL-18) but high levels of cytokines which induce proliferation of Th2 lymphocytes (IL-10, IL-4 and IL-13). (**3**) M2 macrophages engage naïve CD4^+^ T lymphocytes and induce clonal expansion of Th2 cells (**4**) which secrete low levels of cytokines required for disease resolution (and which are produced during infection with acute/non-persistent *Salmonella*) such as IFNγ, TNF-αand IL-17 but high levels of anti-inflammatory cytokines IL-10, IL-4 and IL-13. (**5**) Non-resident (blood-derived) macrophages receive suppressive cytokine signals and are unable to kill intracellular *Salmonellae* which are then (**6**) transported to deeper tissues and may persist in cell types such as gall bladder epithelial cells.

*S.* Typhimurium persistently infects *Slc11a1*^+/+^ mice [4,152,153] and CD4^+^ and CD8^+^ T cells [85] and IFNγ [4] are important in final clearance and IL-17 is also thought to be important [153]. Persistent infection in the spleen is associated with elevated numbers of neutrophils, dendritic cells and macrophages but with no increase in T cells [153,154]. These authors showed that CD8alpha+ DC and Gr-1+ cells (neutrophils) increased in the red pulp. Gr-1+ cells, CD68+ cells and CD11c+ cells, the latter lacking detectable staining for CD8alpha and CD4, accumulated around hepatic blood vessels. *S*. Typhimurium is found to be preferentially associated with anti-inflammatory M2 macrophages during the later stages of splenic infection with the intracellular physiology contributing to bacterial metabolism through PPARdelta-mediated fatty acid metabolism and glucose availability; M2 macrophages were identified by CD301 and IL-4Rα markers [155,156]. The *Salmonella* bacteria persist in splenic granulomas that are populated by CD11b^+^CD11c^+^Ly6C^+^ macrophages reprogrammed from M1 to an M2 phenotype, partially by the bacterial effector protein SteE, which modulates STAT3 activity promoting the alternative M2 phenotype [157]. Reprogramming occurs during the course of infection and is limited by TNF production. CD11b^+^CD11c^+^ macrophages are also characteristic of granulomas in experimental *Mycobacterium tuberculosis* granulomas in primates [158]. One question therefore remains as to what causes the switch and reprogramming from M1 to M2 macrophages during infection and whether there is a temporal change in SteE expression in *S.* Typhimurium. This gene is also present in 18/24 *S*. Gallinarum and 2/4 *S*. Pullorum genomes (A. Berchieri and V. Benevenides, unpublished). Higher bacterial numbers lead to increased IFNγ^+^ CD4^+^ T cells, neutrophils and CD301- granuloma macrophages which produce more IFNγ and inducible nitric oxide synthase (iNOS). Lower bacterial numbers therefore could reduce the stimulation of inflammatory mediators and may lead to the switch from an M1 phenotype to an M2 phenotype to reduce the prolonged damaging effect of the inflammatory response. Metabolism of arginine utilized by macrophages involves iNOS (M1 macrophages) or arginase (M2 macrophages) [159,160]. The expression of iNOS by M1 macrophages metabolises arginine to NO, whereas arginine is metabolised by M2 macrophages to urea and ornithine, and this limits the production of NO [161] (Figure 2). Carriage of *Salmonella* in macrophages from the intestinal tissues to the mesenteric lymph nodes and circulation has been reported (reviewed by Mastroeni et al. [162]. However, it is possible that dendritic cells (DCs) may also play a key role in persistence. DCs are abundant within the sub-epithelial dome of Peyer’s patches and following invasion and possible M cell transport, *Salmonella* bacteria are found within these cells [163]. DCs also phagocytose *Salmonella* by penetrating the epithelial cell monolayer tight junctions and are therefore also able to directly sample the intestinal environment [164]. *Salmonella* is known to inhibit MHC II expression by murine DCs and as such are able to suppress CD4^+^ lymphocyte activation [165,166,167]. DCs have also been shown to transport *Salmonella* [163] and since different DC subsets (which may be immunogenic or tolerogenic) have been detected in intestinal tissue [168,169,170] this raises the intriguing possibility that some DC subsets may be involved in persistence or chronic infection while others may be involved in acute infection.

Transcriptional changes associated with a switch from a predominantly Th1 immune response to a Th2 response have also been recorded during persistent gall bladder infection by *S.* Typhimurium [171,172] characterised by increases in immunoglobulins and transcription of the Th2 transcriptional regulator GATA3 and of IL-4 and Stat6.

### 8.2. S. Gallinarum and Pullorum 

Both *S.* Gallinarum and *S*. Pullorum persist in the presence of high titre specific circulating IgY. In vitro both serovars persist in host macrophages and cause less cell death in comparison to more inflammatory serovars and this may be linked to persistence [168] but the basis of this is unknown. Studies with *S*. Gallinarum and *S*. Pullorum have compared the host response to that induced by *S.* Enteritidis, a taxonomically closely related serovar which drives a strong inflammatory response in vivo and in vitro. This is characterised by high levels of IL-17, IL-12 and IL-18 in macrophages and IFNγ in CD4^+^ T lymphocytes co-cultured in vitro with infected macrophages [173,174]. In the spleen, *S*. Gallinarum induced significantly lower levels of iNOS and IFNγ and consistently lower levels of IL-18 and IL-12 but significantly greater expression of anti- inflammatory IL-10 at day 4 and 5 pi when compared to *S*. Enteritidis. This immune phenotype was associated with transit from the intestinal tissues to the liver by *S.* Gallinarum, not observed following *S*. Enteritidis infection. This immunomodulatory mechanism may facilitate typhoid disease in *S*. Gallinarum-infected chickens [174]. In comparison with *S*. Enteritidis, *S*. Pullorum-infected monocyte-derived macrophages show reduced mRNA expression levels of IL-12α and IL-18 and stimulated the proliferation of Th2 lymphocytes, with reduced expression of gamma interferon (IFNγ) and IL-17 and increased expression levels of IL-4 and IL-13. There was little evidence of clonal anergy or immune suppression induced by *S*. Pullorum in vitro. *S*. Pullorum also increased the levels of expression of IL-4 and decreased the levels of IFNγ in the spleen and caecal tonsil of infected birds. This suggests that *S*. Pullorum is able to modulate host immunity from a dominant IFNγ-producing Th17 response toward a Th2 response [173,175].

As yet, we have not elucidated whether either of these avian serovars become localised in M2 macrophages during chronic infection but the fact that *S.* Pullorum-infected macrophages produce low levels of IL-12α/IL-18 but much higher levels of IL-4/IL-13, suggest that *S.* Pullorum infection alone may induce an M2 phenotype [173,176,177].

As mentioned above the *steE* gene, involved in reprogramming M1 to M2 macrophages is also possessed by a high proportion of *S*. Pullorum and *S*. Gallinarum strains. 

**Conclusion:** The mouse studies with *S*. Typhimurium and chicken studies with *S*. Pullorum indicate that in these two cases, at least, the pathogens modulate the host immune response away from a clearing Th1-type response towards a Th2-type response characterised by reduced IFNγ. In mice this is initiated by macrophage switching from a M1 to M2 phenotype induced by the pathogen. The anti-inflammatory response to *S*. Gallinarum infection was demonstrated in a *Salmonella*-susceptible chicken line rather than in a resistant line in which persistence has been demonstrated previously.

## 9. How Does This Information Apply to *S.* Typhi and the Remaining Typhoid Serovars?

### 9.1. S. Typhi

The vast majority of our knowledge regarding the immune response in human typhoid is accrued from the use of the Ty21a vaccine, or experimentally attenuated *S*. Typhi strains, in human volunteers [178,179,180]. The role of CD4^+^, CD8^+^ and IFNγ in controlling acute human typhoid infections is acknowledged [2,178,179,181]. Subsequent to these latter studies, IL-17 production was also found to be produced by CD8^+^ T cells, which also produced IFNγ [182]. Significant increases in IL-17, CD4^+^ T cells and in vitro IFNγ production were also observed during convalescence from *S*. Typhi [183]. Those studies suggested that in the majority of individuals, *S*. Typhi infection induced a predominant IFNγ response derived from lymphocyte subsets other than Th1. Tregs from acute typhoid patients show higher PD-1 and lower CD27 expression, suggesting that they have higher suppressor activity and lower co-stimulatory activity [184]. Although this is in response to an acute infection as opposed to persistent infections, one study has reported that there are decreased levels of inflammatory mediators (IFNγ and IL-17) in the serum of patients with acute typhoid compared to levels from convalescent patients [183], suggesting that the inflammatory response is inhibited during the acute phase but that this is overcome, leading to reduced clinical symptoms and disease convalescence. In humans, in vitro studies have shown that inhibition of the inflammatory response occurs due to expression of the Vi capsular antigen by *S*. Typhi. This includes reduced opsonisation, phagocytosis, and production of oxidative killing pathways [185] and IL-8 production via inhibition of Toll-like receptor signalling [186]. Vi antigen is not expressed by *S*. Typhimurium but one study has shown that insertion of *S*. Typhi Vi antigen into *S*. Typhimurium down-regulates inflammatory immune responses and promotes production of anti-inflammatory IL-10 in mice, which wild type *S*. Typhimurium were unable to do [187]. This immune phenotype is consistent to that reported by Tang et al. [174] in chickens infected with *S*. Gallinarum, which also had a reduced expression of inflammatory mediators but increased levels of anti-inflammatory IL-10 production.

Transcriptional changes in blood, reported by Thompson et al., [71] indicate that carriers exist in two populations with 7/23 individuals studied showing pattern of raised levels of gene expression more closely resembling post-acute patients and with the remainder showing much lower levels. This latter group included a reduction in lymphocyte numbers, transcripts associated with CD8^+^ cytotoxic T lymphocytes, several neurotransmitter transcripts and glutamate receptor SLC1 A6 found in Kupfer cells [188]. As with all transcriptional analysis it was acknowledged that these changes could equally well reflect cellular changes occurring as a result of infection which would nevertheless be highly significant.

Similar profiles were found in mice persistently infected with *S.* Typhimurium [189]. Although the authors infer *Salmonella*-induced immune suppression [190,191,192], long term evasion of the immune response was also considered [97,193]. 

Proteomic analysis [194] of blood from chronic typhoid carriers compared with healthy individuals indicated increased proprotein convertase, subtilin, furin, haptoglobin and albumin correlated with increases in the relevant mRNA. Albumin and haptoglobin have a role in free radical generation and generation of RNS in chronic inflammation and activate monocyte signalling pathways. Furin has also been shown to act as a TGF-β1 converting enzyme via proteolytic cleavage of the secreted (inactive) form to biologically active TGF-β 1 [95,195] which, in murine *S*. Typhimurium infection is also associated with decreased *Salmonella* numbers in liver and spleen [96]. 

### 9.2. S. Dublin

**Figure 3:**
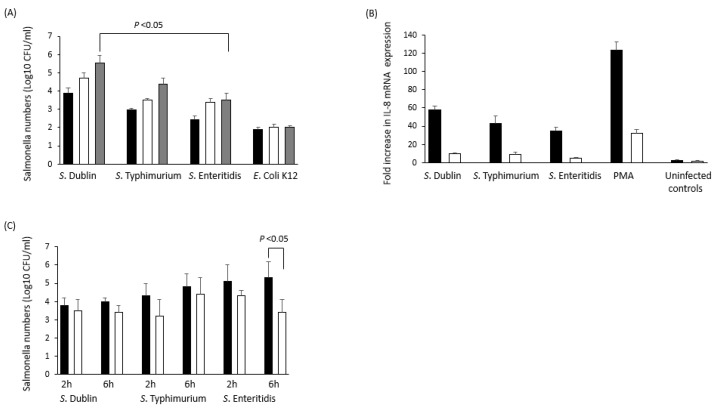
Comparative invasion dynamics and IL-8 production in Bovine MDBK cells and neutrophils infected with *S*. Dublin, *S*. Typhimurium or *S*. Enteritidis. *S*. Dublin invade MDBK cells in significantly greater numbers than *S*. Enteritidis after 24 h post-infection (**A**). After 2 h post-infection, *S.* Dublin also induces significantly increased IL-8 mRNA expression when compared to *S*. Enteritidis (**B**). Lower uptake of *S.* Dublin by neutrophils occurred compared to *S.* Enteritidis but this was not significant and the addition of IFNγ reduced intra-cellular survival in *S.* Enteritidis-infected neutrophils only after 6 h post-infection (**C**). IL-8 expression analysis was performed according to the Pfaffl method and using 18s RNA as a reference. MDBK data was obtained from a mean of 5 replicate experiments performed in triplicate. Neutrophils were obtained from 5 healthy individual, 3-year-old Holstein Friesian cows and triplicate experiments were performed from the blood of each cow. Error bars show standard deviation from the mean and linkage bars show significant difference at *P* = 0.05, as determined by a one-way analysis of variance (ANOVA). (**A**) Black bar = 2 h post-infection; White bar = 6 h post-infection; Grey bar = 24 h post-infection. (**B**) Black bar = 2 h post-infection; White bar = 6 h post-infection. (**C**) Black bar = Cells cultured without IFNγ; White bars = Cells cultured with recombinant bovine IFNγ (100 μg/mL). PMA = Phorbol myristate acetate (10 μg/mL).

There are few studies of the immune parameters associated with *S.* Dublin persistence. A study by Deng Pan et al. [118] reported that in 3-week-old calves, *S.* Dublin induced lower levels of TNFα and IL-12 than *S.* Typhimurium or *S*. Enteritidis, which are strongly inflammatory serovars, but a greater level of IL-8 which is a neutrophil chemoattractant and therefore an inflammatory chemokine [196]. Our studies (Foster et al., unpublished) have also shown that *S*. Dublin is able to invade Madin Derby Bovine Kidney (MDBK) cells in significantly higher numbers than *S*. Enteritidis and at higher levels (but not significantly) when compared to *S*. Typhimurium, although significantly higher (*P* < 0.05) numbers were recovered from S. Dublin-infected MDBK cells after 24 h pi (Figure 3). Similarly, we found that *S*. Dublin significantly increased IL-8 mRNA expression (neutrophil chemoattractant) when compared to *S*. Enteritidis at 2 h pi and although IL-8 expression was increased above expression levels in MDBKs infected with *S*. Typhimurium this was not significant. This indicates that the greater invasion of epithelial cells by *S*. Dublin in vitro induces greater IL-8 expression but we found that fewer *S*. Dublin were phagocytosed by bovine neutrophils at 2 and 6 h pi when compared to either *S*. Typhimurium or *S*. Enteritidis. Furthermore, the addition of IFNγ to these cultures only caused a significant decrease (*P* < 0.05) in intra-cellular *S*. Enteritidis after 6 h pi (Figure 3).

Therefore, these studies suggest that the ability of *Salmonella* to evade or resist innate immune killing pathways may not be relevant to the establishment of carrier status, since *S.* Dublin and *S*. Typhimurium show similar profiles but only *S.* Dublin induces carrier status.

**Conclusion:** The few studies with *S.* Typhi suggest that convalescents which enter the carrier state, showing true persistent infection, are associated with transcriptional and proteomic changes in the blood which may be associated greater bacterial survival and an anti-inflammatory response. Little can be said of *S*. Dublin since although in vitro and in vivo work indicates greater survival of *S.* Dublin in macrophages compared with *S.* Enteritidis and increased IL-8 and reduced IL-12 and TNFα, it is currently impossible to say whether this relates solely to early stages of acute infection rather than persistence.

## 10. Can Anything Be Done to Reduce the Impact of Persistent Infection by Remodulating the Immune Response?

At an empirical level there has been relatively recent interest in remodulation of the host immune response away from that driven by pathogens to one which benefits the host. Such studies have involved bacterial and parasitic infections and it has been suggested that it is possible to re-modulate the immune response to the benefit of the host. In human leprosy, intradermal IFNγ administration has been shown to change local infection from lepromatous to tuberculoid leprosy, with increases in the numbers of CD4^+^ T-cells and reductions in bacterial numbers in dermal biopsies [197,198]. IL-12 administration, which is produced by innate cells such as macrophages and stimulates generation of IFNγ-producing Th1 lymphocytes, has also been shown to cure mice infected with *Leishmania major* [199]. Finkelman et al., [200] were able to modulate the mouse response to *Nippostrongylus braziliensis* infection, away from a Th2 dominant response, characterised by IL-3 and IL-4 production, by parenteral administration of IL-12. *In ovo* administration of chicken IFNγ is being considered for protection against a number of avian pathogens including chicken anaemia virus and for its adjuvanticity in vaccine formulations [201,202]. Barrow [203] showed that intravenous administration of a single large dose of recombinant chicken IFNγ during persistent *S*. Pullorum infection led to a reduction in the total number of infected spleens: 4/18 (22%) spleens positive for *S*. Pullorum in the IFNγ-treated animals and 7/13 (54%) in the untreated controls (*P* < 0.01). In another study, recombinant chicken IFNγ was also able to enhance NO production in avian peripheral blood monocyte-derived macrophages and reduce the intracellular replication of *S*. Typhimurium and Enteritidis [204]. Practically, cytokine therapy is unlikely to be considered for *S*. Pullorum in susceptible commercial chickens and is unlikely to be effective against *S*. Gallinarum, which shows persistence in *SAL1*^R^ chickens similar to *S*. Typhimurium in *Slc11a1*^+/+^ mice which requires IFNγ activity [4]. However, it is conceivable that it may have some application in therapeutically reducing persistence in the liver and spleen in human typhoid carriers or may reduce gall bladder infection if administered during acute infection. 

**Conclusion:** There is clearly scope for further investigation of administration of cytokines to modulate the nature of the immune response or perhaps to administer vaccines therapeutically [205,206] which may have the same effect in reducing persistent infection of the spleen and liver caused by typhoid *Salmonella* serovars.

## 11. Conclusions

Persistent infection caused by the typhoid *Salmonella* serovars remains only partially understood with serovars such as *S*. Typhi, *S*. Typhimurium, *S*. Gallinarum and *S*. Pullorum but is poorly understood and also poorly described for the economically important livestock pathogens *S*. Dublin and *S*. Abortusovis. An important aspect of persistence appears to be modulation of the immune response away from a protective Th1-type response to an anti-inflammatory or Th2-type response which suppresses tissue clearance and may involve *Salmonella*-driven M1 to M2 macrophage switching. What induces this change and the microbial factors involved in the induction have not been fully identified. Re-modulation of the immune response through cytokine therapy or therapeutic vaccination may present a way forward to resolve these infections which continue to cause problems in public and animal health.

## Data Availability

Not applicable.
